# Supplementary material for: The molecular architecture of the meiotic spindle is remodeled during metaphase arrest in oocytes
Source: J Cell Biol. 2019 Jul 5;218(9):2854–64. doi: 10.1083/jcb.201902110 (PMC6719438; doi:10.1083/jcb.201902110)
Supplement: Supplemental Materials (PDF) [file JCB_201902110_sm.pdf]

Supplemental material

Costa and Ohkura, https://doi.org/10.1083/jcb.201902110

A

LOCALISATION IN OOCYTES		
SPINDLE PROTEINS CHANGED	Early (stage 13)	Late (stage 14)
Sentin	All over the spindle	Spindle poles
Kinesin-5/KLp61F	Spindle poles	Spindle equator
Augmin/Dgt6	Weakly to spindle poles	Strongly to spindle poles
TPX2/Mei-38		
MKlp1/Pavarotti	Not localised / Weakly all over the spindle	Spindle equator
PRC1/Feo		Spindle equator
TACC	Spindle poles + foci on the spindle	Spindle poles
XMAP215/Msps		
ASPM/Asp	Spindle poles (57%)	Spindle poles (100%)
(at kinetochores)		
Polo kinase	Kinetochores and spindle	Weakly to spindle
CycA	Kinetochores	Not localised
Mad2	Kinetochores	Not localised
Clasp	Kinetochores	Kinetochores and KT-microtubules
Rod	Kinetochores	Kinetochores and KT-microtubules
UNCHANGED	both stages	
Incenp	Spindle equator and kinetochores	
MKlp2/Subito	Spindle equator	
EB1	All over the spindle	
MCAK/Klp10A	Spindle poles and kinetochores	
Kinesin-8/Klp67A	All over the spindle and kinetochores	
Cyclin B	Spindle equator	
Kinesin-4/Klp3A	Around the chromosomes	
Hurp/Mars	Spindle poles	
Patronin	All over the spindle	
Map205	Not localised	
Nedd1/Grip71	Not localised	

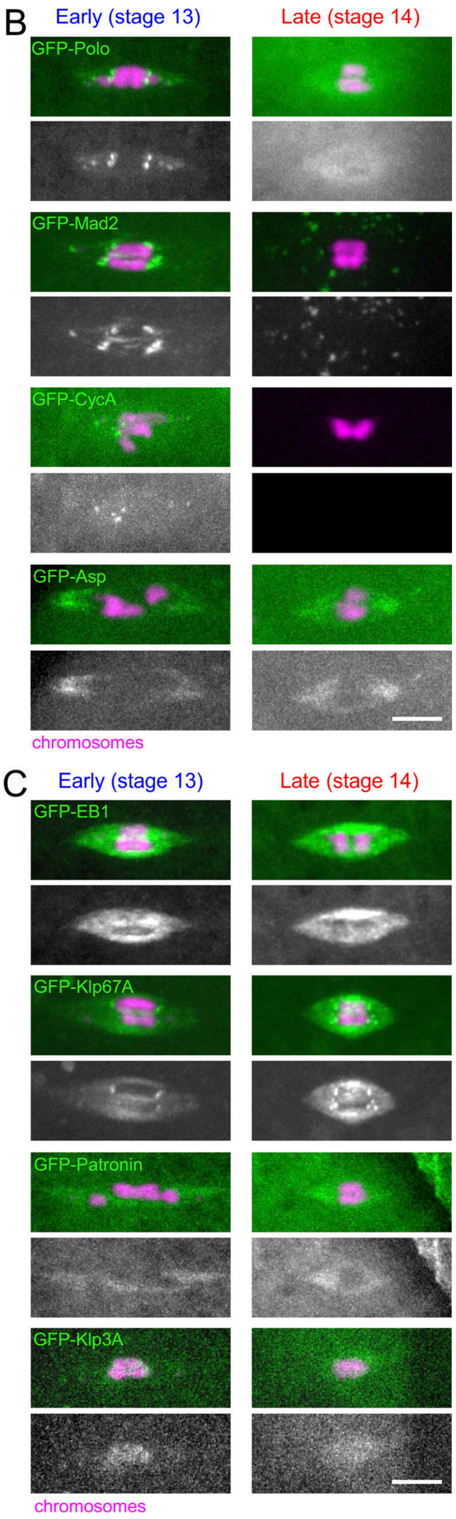

Figure S1. **Many spindle proteins change their localization during arrest. (A)** List of spindle proteins examined in this study. **(B)** Representative images of proteins that change localization from kinetochores to kinetochore microtubules or are diffused in oocytes. From top, a total of 17, 31, 47, and 14 stage 13 oocytes and 23, 35, 52, and 52 stage 14 oocytes were observed. Polo, Mad2, and Cyclin A were concentrated on kinetochores (strong dots), and Polo and Mad2 also localized weakly to the spindle in stage 13. These localizations were lost in stage 14. **(C)** Spindle proteins that do not change their localization between early and late oocytes. A total of 10, 12, 13, and 13 stage 13 oocytes and 21, 35, 21, and 7 stage 14 oocytes were observed. Bars = 5 µm.

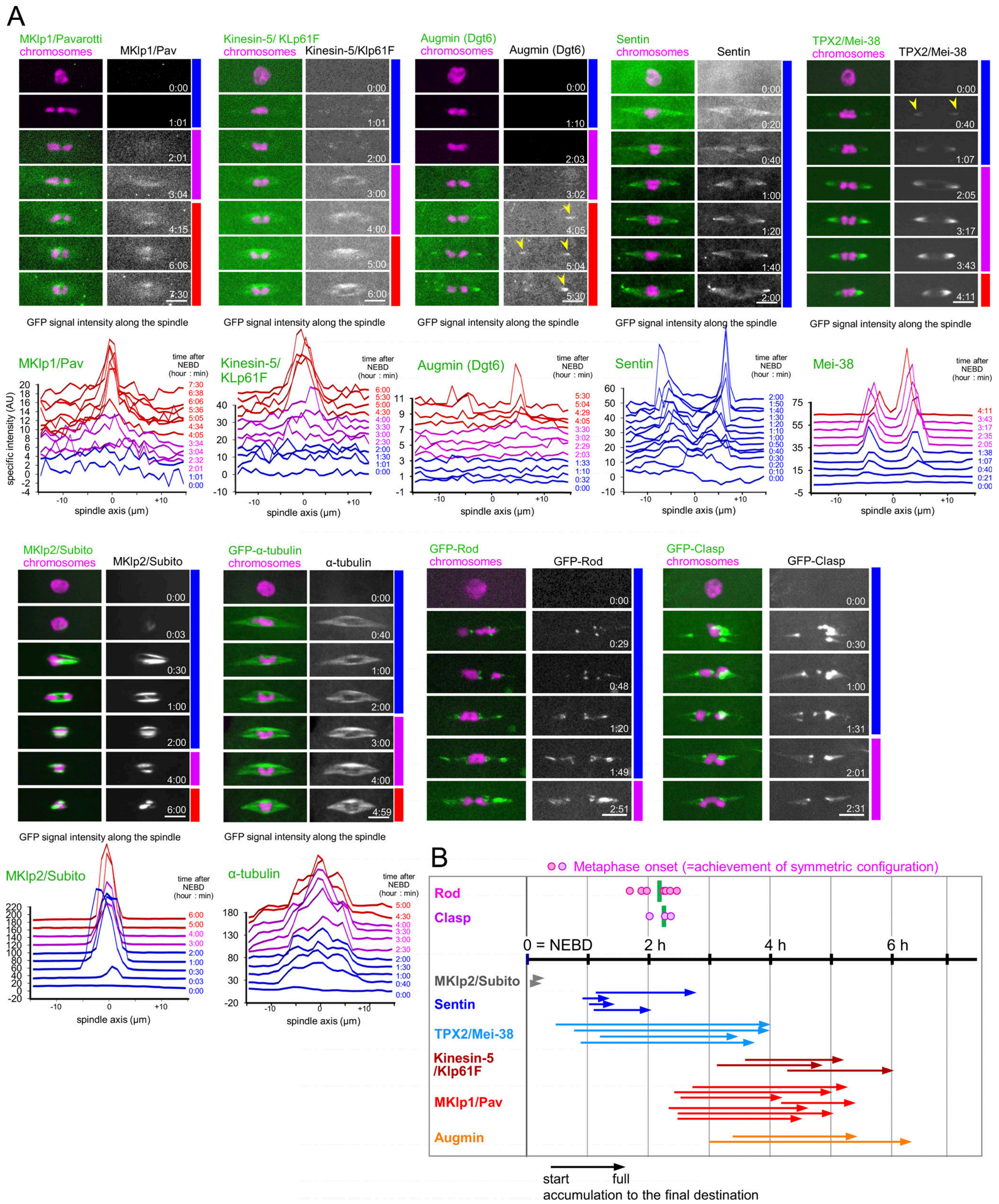

Figure S2. **Time-lapse imaging of spindle proteins in oocytes. (A)** Time-lapse images of the spindle in oocytes expressing a GFP-tagged protein and Rcc1-mCherry, quantified as in Fig. 2. For GFP-Sentin, the time-lapse images published in Głuszek et al. (2015) were reanalyzed for comparison. The arrowheads indicate accumulation of Augmin or TPX2/Mei-38 to the spindle poles. Bar = 5 µm. **(B)** Timing of metaphase onset and localization changes of spindle proteins estimated from time-lapse live imaging.

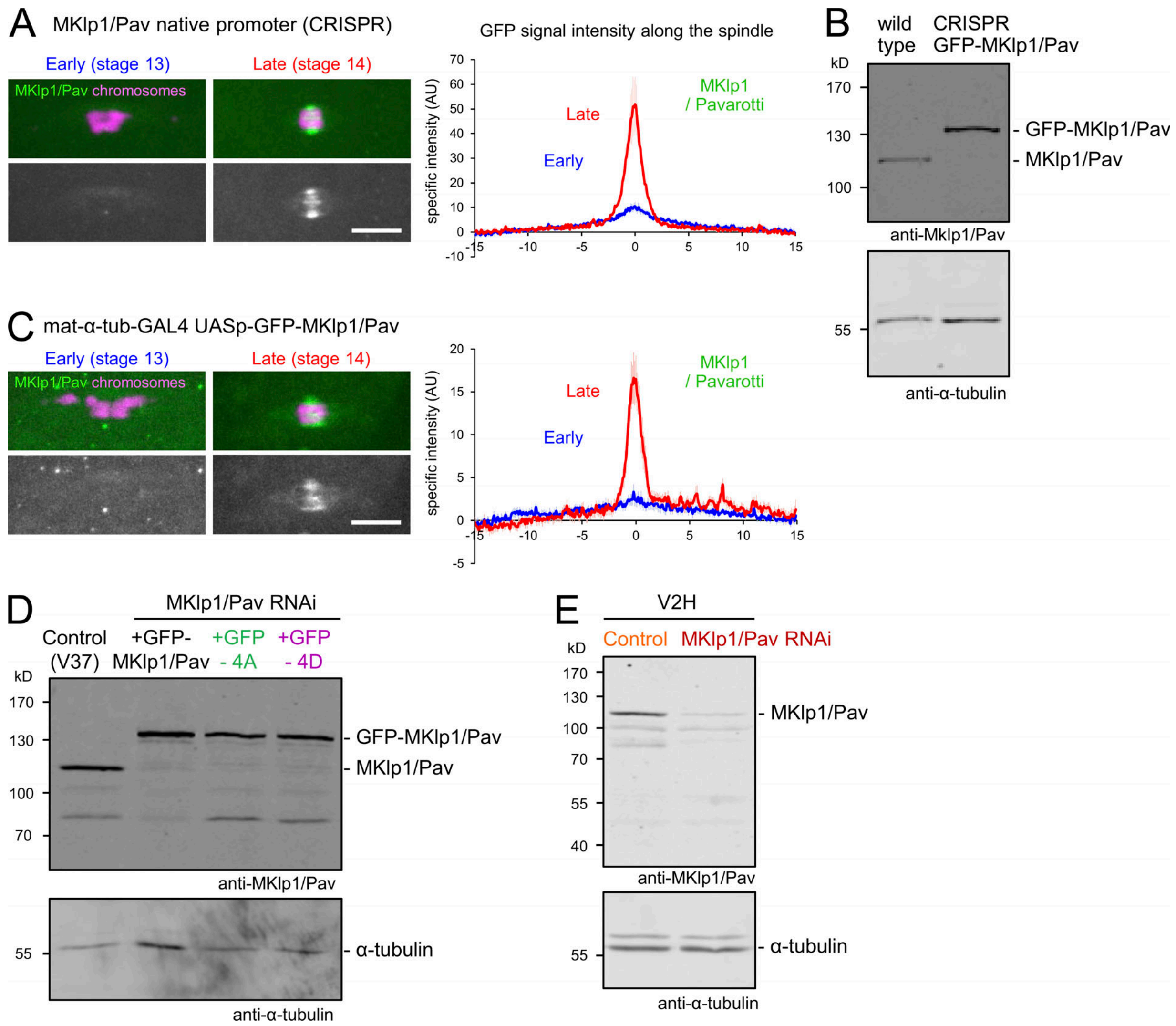

Figure S3. **Expression and localization of GFP-MKlp1/Pav in oocytes. (A)** GFP-MKlp1/Pav expressed under the MKlp1/Pav native promoter at the native locus, together with Rcc1-mCherry, in CRISPR-generated lines. *n* = 11 each. **(B)** Immunoblot analysis of ovaries shows expression of GFP-tagged MKlp1/Pav and loss of the endogenous MKlp1/Pav in the CRISPR-generated line. **(C)** GFP-MKlp1/Pav expressed under the UASp promoter driven by GAL4 under the maternal α-tubulin promoter, together with Rcc1-mCherry. *n* = 13 each. **(D)** Immunoblot analysis of ovaries shows comparable protein amounts and sizes of GFP-Mklp1/Pav variants expressed from transgenes and depletion of the endogenous protein achieved by RNAi. **(E)** Immunoblot analysis of ovaries shows partial depletion of MKlp1/Pav in stage 14 oocytes expressing shRNA driven by a weaker GAL4 driver (V2H). Bars = 5 µm. AU, arbitrary units.

Reference

Głuszek, A.A., C.F. Cullen, W. Li, R.A. Battaglia, S.J. Radford, M.F. Costa, K.S. McKim, G. Goshima, and H. Ohkura. 2015. The microtubule catastrophe promoter Sentin delays stable kinetochore-microtubule attachment in oocytes. *J. Cell Biol.* 211:1113–1120. https://doi.org/10.1083/jcb.201507006
